# *Kikiskawâwasow* - prenatal healthcare provider perceptions of effective care for First Nations women: an ethnographic community-based participatory research study

**DOI:** 10.1186/s12884-016-1013-x

**Published:** 2016-08-11

**Authors:** Richard T. Oster, Grant Bruno, Margaret Montour, Matilda Roasting, Rick Lightning, Patricia Rain, Bonny Graham, Maria J. Mayan, Ellen L. Toth, Rhonda C. Bell

**Affiliations:** 1 Department of Medicine, Research Transition Facility, University of Alberta, Edmonton, AB T6G 2V2 Canada; 2 Department of Agricultural, Food and Nutritional Science, Li Ka Shing Centre for Health Research Innovation, University of Alberta, Edmonton, AB T6G 2R3 Canada; 3 Samson Cree Nation, Maskwacis, AB T0C 1N0 Canada; 4 Louis Bull Cree Nation, Maskwacis, AB T0C 1N0 Canada; 5 Ermineskin Cree Nation, Maskwacis, AB T0C 1N0 Canada; 6 Maskwacis Health Services, Maskwacis, AB T0C 1N0 Canada; 7 Faculty of Extension, Enterprise Square, University of Alberta, Edmonton, AB T5J 4P6 Canada

**Keywords:** Indigenous population, Qualitative research, Prenatal care, Community-based participatory research, Health care providers

## Abstract

**Background:**

Pregnant Indigenous women suffer a disproportionate burden of risk and adverse outcomes relative to non-Indigenous women. Although there has been a call for improved prenatal care, examples are scarce. Therefore, we explored the characteristics of effective care with First Nations women from the perspective of prenatal healthcare providers (HCPs).

**Methods:**

We conducted an ethnographic community-based participatory research study in collaboration with a large Cree First Nations community in Alberta, Canada. We carried out semi-structured interviews with 12 prenatal healthcare providers (HCPs) that were recorded, transcribed, and subjected to qualitative content analysis.

**Results:**

According to the participants, relationships and trust, cultural understanding, and context-specific care were key features of effective prenatal care and challenge the typical healthcare model. HCPs that are able to foster sincere, non-judgmental, and enjoyable interactions with patients may be more effective in treating pregnant First Nations women, and better able to express empathy and understanding. Ongoing HCP cultural understanding specific to the community served is crucial to trusting relationships, and arises from real experiences and learning from patients over and above relying only on formal cultural sensitivity training. Consequently, HCPs report being better able to adapt a more flexible, all-inclusive, and accessible approach that meets specific needs of patients.

**Conclusions:**

Aligned with the recommendations of the Truth and Reconciliation Commission of Canada, improving prenatal care for First Nations women needs to allow for genuine relationship building with patients, with enhanced and authentic cultural understanding by HCPs, and care approaches tailored to women’s needs, culture, and context.

## Background

In the Cree language, *kikiskawâwasow* means “she is with child”. Pregnancy is a crucial period in the health trajectory of a family. In addition to acute impacts, the fetal circumstances in utero substantially affect future risk of developmental difficulties/challenges and adult diseases [1]. A growing body of perinatal research has detailed considerable global health disparities experienced by Indigenous women and their infants compared to non-Indigenous populations [2]. Similar disparities have been shown among Canada’s most populous Indigenous group^1^, the First Nations, in Alberta [3, 4]. Moreover, among First Nations women fertility/birth rates are significantly higher and inadequate prenatal care occurs more often than in non-First Nations women [5, 6]. Specifically, pregnant First Nations women in British Columbia (Canada) were less likely to have early ultrasonography, to have at least four antenatal care visits, and to undergo labour induction after prolonged pre-labor rupture of membranes or at post-dates gestation compared to non-First Nations women [6]. Improved care and a greater understanding of how to effectively work in this context is needed.

Frequent dissatisfaction with prenatal healthcare providers (HCPs) has been shown in qualitative work with Indigenous women [7–11]. On the other hand, from the perspective of prenatal HCPs, lack of time and system-level resources are often described as barriers to appropriate care for Indigenous women [12, 13]. This report is part of a community-based participatory research (CBPR) project in collaboration with a large Cree First Nations community in Alberta. It builds from an ongoing community engagement process that has taken place over several years. Our objective was to explore the characteristics of effective care with First Nations women from the perspective of HCPs that service a large First Nations community in Alberta. For our purposes, effective care essentially referred to care that contributed to healthier pregnancies and better prenatal outcomes. This work is one piece of the overarching ENRICH study (www.enrich.ales.ualberta.ca), which aims to improve maternal health in pregnancy and postpartum within Alberta.

## Methods

### Study design and setting

This study was carried out in collaboration with the Cree community of Maskwacis, Alberta (collectively made up of Samson, Louis Bull, Montana, and Ermineskin First Nations bands; includes Pigeon Lake reserve), with which we have pre-existing research relationships. Our approach was informed by both ethnography and CBPR. The foundation of CBPR is true partnerships between the researcher and community members to resolve significant social and/or health problems [14]. CBPR considers community members as experts on their own experience, carrying knowledge and skills that are valuable to the research process [15]. Ethnography is a qualitative method that is increasingly being adapted in healthcare settings to view and understand perceptions, beliefs, shared meanings, values, and/or practices in the context in which they occur [16, 17].

Our community partners voiced a need to improve the pregnancy-related health of women from the community, and desired a CBPR collaboration. After an initial year and a half of engagement activities within the community by Oster (e.g. developing strong relationships and friendships, volunteering at the health centres, attending community events, participating in sub-committee meetings, taking part in ceremonies and cultural events, assisting with ongoing research projects in the community, making presentations when asked, etc.) a research partnership was formed through the development of a Community Advisory Committee (CAC). Childbearing women from Maskwacis were involved in the engagement activities to help better understand the community context and to inform the CAC meetings. The CAC acted as our main community collaborator and consisted of a fluid group of community members, Elders, and staff from the community health/social services departments. Through regular collaborative meetings, the CAC provided guidance and advice to all involved and devoted time to jointly design the research protocol, contribute to the interpretation of the data, and approve all dissemination pieces. Several research objectives were established, including understanding the perceptions of HCPs (current study), fathers (parallel study), as well as Elders and women of childbearing age (parallel study). The CAC partnership agreed ethnography would be the most appropriate approach to achieve the research objectives as it invokes a collaborative process that seeks to involve participants in the research process, and is both compatible with and complimentary to CBPR [16].

Maskwacis is a rural community (not remote) approximately 90 km south of Edmonton, Alberta, within the area of Treaty Six. The registered population of the four bands is 16,004. Pregnant women must travel to neighboring off-reserve towns and cities for prenatal care, with the majority receiving care at a single Primary Care Network associated with delivery at a Hospital that is approximately 15 km from the community town site. However, some pregnancy-related care and most postnatal care is delivered within the community (e.g. prenatal classes, diabetes clinic which sees women with diabetes complicating pregnancy, home care team, immunizations and postpartum care, etc.) and in other surrounding communities.

### Ethics

Ethical approval by the University of Alberta Research Ethics Board was obtained. Community approval from key stakeholders and Elders was also obtained. Community members were involved at all stages of this project, with the aim of shared power, equitable resourcing and mutual understanding. The CAC also provided ethical assistance and ensured the research progressed in a culturally appropriate manner. A research agreement was jointly developed with the CAC. Throughout the study there was capacity building within the community through the involvement of community members whenever possible (e.g. hiring of community members as research assistants, locally sourcing any research-related expenditures, etc.).

### Sample

HCPs (nurses, physicians, dietitians, mental health therapists) within Maskwacis and neighboring non-Indigenous communities were recruited using purposeful sampling. We wanted to attain a sample that was proportionately representative of those providers who deliver prenatal care to women from the community. Midwifery is generally uncommon in the province of Alberta, and to our knowledge there are no midwives working in this community. We recruited HCPs that worked regularly with pregnant and postpartum women from Maskwacis and provided health-related or comprehensive care to women during the childbearing year. We sought to have a mixture of Indigenous and non-Indigenous participants, and on-reserve and off-reserve participants. All of the participants had been working with women from Maskwacis for a minimum of one year. An information letter detailing the study was reviewed with HCPs who agreed to participate, and written informed consent was provided prior to interviews.

### Data generation

The data were generated from June to December 2015 via one-to-one semi-structured interviews at a mutually selected location. The CAC partnership agreed that representative voice of the participants was the aim of the data generation activities and felt participant observation (often a core data collection method of ethnography) may alter the participant’s behavior. Since participant observation was also agreed to be too time consuming for the project goals, this approach was not utilized. The interviews were conducted by Oster and Bruno in English, lasted approximately 45–60 min, and were audio-recorded and transcribed verbatim. Bruno is of Indigenous descent. Interviews were conversational and open-ended questions were asked to prompt discussion (e.g. what kind of advice would you give another HCP that is working with pregnant Indigenous women?). It was our intent to highlight and understand positive practices and not to ‘interrogate’ HCPs or set an over-critical atmosphere for the interviews. The participants were encouraged to focus solely on prenatal care and their Indigenous pregnant patients.

### Data analysis

Data were analyzed together by Oster and Bruno concurrently with data generation using qualitative content analysis [16] and Atlas.ti qualitative computer software. Briefly, transcripts were read and re-read, and coded to determine persistent concepts that were grouped in categories. The categories were again re-read (and re-organized if necessary), and were then described in-depth. The categories were considered together to determine if/how they were related and to identify common themes in all of the data. Initial findings were discussed with the CAC and members of our research team, and feedback was sought from both of these groups. Feedback helped refine the categories and findings, and reduce redundancy. Data collection and analysis ceased upon data saturation, when no new information or insight emerged, and when the categories were well defined.

### Rigor

Strength and rigor were achieved by regularly consulting the CAC and by abiding with the approaches described by Whittemore, Chase, & Mandle [18] and Milne & Oberle [19] to achieve authenticity, credibility, criticality and integrity of data collected. Oster and Bruno kept reflective personal journals throughout the entire research process to allow for reflection and critical appraisal of the information collected. Participants were provided with a draft of the findings and invited to provide feedback to enhance the trustworthiness and correctness of the data, eight of which did.

## Results

A total of 12 participants were needed to reach data saturation. The average age of participants was 42 years. Six of the participants worked within the community and six worked off-reserve. Three of the 12 participants were Indigenous, which is representative of the HCPs available to pregnant women in this community and in the surrounding communities (Indigenous HCPs are the minority). Seven of the participants were nurses, two were physicians, two were dietitians, and one was a mental health therapist.

Three core categories that were highly interrelated and consonant with one another were identified from the analysis. The first category, Relationships and Trust, was foundational to the other two categories of Cultural Understanding and Context-Specific Care, and all three were identified as critical to effective prenatal care with First Nations women. Moving beyond the mainstream patient-provider relationship and the healthcare system as whole was also a central theme to the categories. An in-depth description of each category is presented below.

### Relationships and trust: “It’s all about relationships”

The participants felt strong relationships and trust with their Indigenous patients was crucial to effective prenatal care and ultimately leads to healthier pregnancies. Going beyond the usual patient-provider relationship by taking more time to get to know patients and including personal investments was necessary according to the participants to achieve genuine relationships and trust.

Building authentic relationships and trust with patients, particularly at the first visit, was uniformly identified by the participants as the basis of effective care. Participants felt such relationships contributed to the support networks of patients and helped reduce fear of the healthcare system that may have arisen from previous negative experiences, judgment, and discrimination. Building such relationships and trust was seen as making it more likely those patients would attend their appointments and be receptive to health messages/education. This was believed to result in better pregnancies: “We need to spend a lot more time building relationships if we are going to have better health outcomes.” In many instances this involved getting to know patients over numerous clinic visits and having conversations about whatever that patient was dealing with that day, as one participant articulated:

I think the biggest thing is just trying to create that relationship with the moms. I know they are hesitant accessing health care. They may have had poor or bad experiences with other nurses… We don’t know that patient’s story. That’s probably the first thing you could do is ask them. Not about why they’re there at their appointment, but to try to talk to them and create a conversation. Ask them about who they are, about their family, and really try to get to know them… Really just being open to whatever they bring to us and talk about that first.

Another participant went on:

Never assume anything. Get to know that person. I always ask them to tell me their story, you know, what’s going on. I don’t go in with a paper and pen and start ticking off the baby’s vital signs… I listen to their story and that’s the first thing in gaining trust, because it might not be about having the baby or the physical labour. It might be that she had a fight with her boyfriend and she’s really, really depressed and she could care less about the baby right now… The moms have my cell number, they can text me anytime.

Developing relationships of trust often entailed transcending the typical formal patient-HCP relationship, moving more towards personal investments and advocating for patients. This necessitated “treating them like a friend”, “being respectful”, “acknowledging their victories”, “being open and honest”, “getting to know the names and lives of these women”, “asking open-ended questions”, “being non-judgmental”, “using a lot of humor”, “empathy”, “focusing on the positives”, “sharing with them”, “building rapport”, “being a little bit vulnerable”, “putting our egos aside”, “being equal”, and “just shutting up and listening a little bit”, to name a few. One participant expressed the need for meaningful relationships beyond the conventional patient-HCP relationship:

It’s about building one relationship at a time. It’s about being a person. I don’t think anybody thinks of me as a health professional… You have to be genuine. You can’t be faking because that would be totally insincere and obviously wrong. But if you feel something is appropriate, say it. Like ‘oh my gosh you are so beautiful.’… But I think sometimes professionals might be way too much to the book… You are not supposed to hug people apparently. Oh well, you know what? If they need a hug, they are getting one. And yeah, you are not supposed to use affectionate terms. You know what? Some of my clients are my ‘dears’. Yep, some of them are my ‘sweeties’.

Some participants described the need for changing system priorities to focus more on building relationships: “the organization likes to see numbers and they don’t necessarily look at relationships, but until you actually build a relationship and you build that trust you’re never going to get anywhere with that client.” Establishing meaningful relationships and interaction with patients also required finding “the right staff”. One participant explained: “They need to ask themselves ‘why are they working with First Nations people?’ If you don’t know why, then maybe this isn’t a good fit for you.” Participants felt HCPs that are open, sincere, compassionate, willing to go “that extra mile”, and able to understand the many issues their First Nations patients may be facing, are better suited to working with this population and are more likely to find such work enjoyable and an opportunity for mutual learning.

### Cultural understanding: “if you have never been out there, you really have no idea”

According to the participants, Cultural Understanding specific to the community served is vital to trusting relationships and effective prenatal care, and entails not only an understanding of Indigenous culture but their histories and context as well. Enhanced cultural understanding was sought by all of the HCPs and was thought to be more impactful if it arose via real experiences and learning from patients rather than relying only on formal cultural sensitivity training alone.

Participants believed that better cultural understanding for HCPs was an important part of enhancing effective care of First Nations women and would further strengthen patient-provider relationships, lessen patient fear of the healthcare system, and provide a more welcoming environment for patients. The participants also felt enhanced cultural understanding would reduce provider frustrations, reduce provider stigmatization and discrimination of First Nations patients, further develop provider compassion and awareness, and encourage more appropriate care recommendations. Taken together, the participants believed this would decrease the number of missed appointments and improve care outcomes. One participant felt willingness to develop a deep cultural understanding should be “a prerequisite to anyone that’s working with First Nations people on a regular basis.”

All of the participants identified that they had an individual need for greater cultural understanding (even the Indigenous participants), and some longed to spend more time with Indigenous people and Elders to learn. The participants were interested and intrigued to know more about First Nations culture and history, and felt they would be more effective HCPs as a result. However, many described not knowing how or where to receive such knowledge or training, and were afraid of feeling “stupid or judged” or “offending someone” by not knowing the appropriate protocol to ask for cultural knowledge. The participants explained that although positive attempts have been made to improve the cultural sensitivity of the healthcare system in Alberta and in Canada, the system remains “nowhere near adequate.” While receiving their health discipline training, the participants learned very little, if anything at all, about Indigenous culture or peoples. Moreover, most of the participants had never received any formal cultural sensitivity training despite the majority of their current work being focused on First Nations patients: “My entire training on cultural sensitivity was five minutes with a fellow from Health Canada. I have never had formal cultural sensitivity training.” Another participant elaborated:

It wasn’t enough. There were these bits and pieces here and there, kind of scattered all over. But no, not near enough. I truly believe that all health care providers, no matter what discipline they are in, need their own individual course that is a part of their program. A component that addresses Aboriginal health issues… The faster that happens, the more prepared health care providers will be to deal with them and the issues that we face.

Those participants that remember receiving some sort of training regarding cultural understanding, such as through manuals/documents or short presentations, described it as largely ineffective. However, as a result of having formed good, trusting relationships with their patients, some participants had learned from their patients and attained a greater awareness and appreciation of patients’ context and culture: “I think it’s important to ask, and lots of people are willing to tell you if you take the time to ask them.” Also, authentic experiences with Indigenous people and Elders, ideally within communities, tended to have a much more profound effect on their cultural awareness and understanding:

We had an Elder come in and we did a big circle, and she told us her story. We were all crying and we had no idea. It was like we were hit by a truck…I also took a couple of courses with an Aboriginal teacher (that took place within) an old residential school… They can teach you things in books, but going into the school and walking past the morgue and hearing stories, that was a huge eye-opener of what people lived through and what lots of families lost. It definitely gave me a lot more compassion… Cultural sensitivity training in a classroom is great, but I think it’s those relationships with community members, hearing someone tell their story is way more impactful.

Cultural understanding encompassed not just an appreciation and grasp of cultural practices, but also knowledge of the ongoing impacts of colonization, and the vulnerability and numerous challenges some patients may encounter: residential school legacy,^2^ racism, historical trauma, cultural loss, addictions, family violence, mental health issues, diabetes, crowded homes, food insecurity, poverty, poor support, limited opportunities, and lack of reliable transportation to name a few. Subsequently, HCPs were better able to understand why some First Nations women missed appointments (which was consistently cited as the most important barrier to prenatal care), as one participant described: “When you’re dealing with all those social determinants of health, then attending appointments is not a priority. And I don’t think it should be a priority either.” Some participants felt it necessary to openly acknowledge the negative impact of colonization to encourage dialogue, stronger relationships, and cultural understanding.

### Context-specific care: “It is not one size fits all prenatal care”

Context-specific care, although not always possible, was perceived by the participants as a key piece of effective prenatal care. They felt context-specific care requires both individual HCPs and the systems in which they work to adapt a more flexible, all-inclusive, and accessible approach that meets specific needs of their Indigenous female patients.

Providing more context-specific care at the individual HCP and health care system levels mitigated some of the barriers to care specific to First Nations women according to the participants. For instance, participants spoke of recent changes in their organization to provide a more open-door style of care, where pregnant women are able to receive care on a walk-in basis if they have not made an appointment, and the ability and willingness of staff to “stay open later just to accommodate certain people.” In recognizing that missed appointments are common for a variety of reasons, participants expressed striving for “all-inclusive”, “multi-disciplinary” and “single-session” care that offers “full service for prenatal care services” to make it easier for their patients. Participants also felt it is important to “meet them where they are at”, as one participant explained: “Just work with the person in front of you and whatever is going on in their life. Working with people as if it might be the only time you see them.”

Moving beyond current standard prenatal care was key. For example, one participant explained that she sometimes met with clients at alternative locations: “There is one girl that had just had a baby and wanted to go back to school. So I actually went to the school and I did the visitation there and it was really good.” Other ideas for context-specific care that had either worked well in the past or were currently being advocated for included: Elder support/mentoring, lactation consultants, menopause programs, midwifery programs, and childcare (ideally accessible within the same facility). Further, improving the coordination of care between the many different clinics and hospitals in the area (both on and off-reserve) was viewed as vital to improving overall care. Participants regularly spoke of their patients struggling to navigate an often overwhelming and complex system.

Context-specific care with First Nations women called for a different ‘bedside manner’ than what many of the participants were used to and that often included genuine listening and counselling. Participants again spoke of the need for equality with patients rather than usual patient-provider relationships that often imply an imbalance of power. Accordingly, HCPs should “actually explain how things work instead of just telling people what to do.” Further, one participant explained:

I’m not going to tell them like ‘this is what you have to do’, because that approach never works… You have to give control back to people, because when they feel like they’re out of control already in their lives, its not going to be helpful when they come to our doors and they don’t have any control at all.

Providing context-specific care, while identified as essential by many participants, was not always attainable within the healthcare system. Participants portrayed an often inflexible mainstream system built on structure, scheduled appointments, rules, policies, time management, order, etc. that did not necessarily meet the needs of First Nations women: “Our biggest thing is that we are kind of pigeon-holed into time.” Another participant provided an additional poignant example: “In the past I have tried to advocate for clients getting their own homes. I got slapped on the wrist because we’re not supposed to be political, when Aboriginal health is one of the most political things in Canada.” Staff turnover was also described as a barrier to providing appropriate care and to women attending appointments, as the participants felt women need to see “familiar faces every time they come in.” Participants expressed feeling “frustrated”, “irritated”, “guilty”, “limited”, “discouraged”, and like their “hands are tied” when unable to provide care they felt essential for First Nations women due to system barriers. As was the case for building relationships and trust, providing context-specific care was limited not only by the ‘system’ but also by the individual as it requires the right staff that are willing to adapt their care approach.

## Discussion

We sought to explore the characteristics of effective care with First Nations women from the perspective of HCPs in a large First Nations community in Alberta. Relationships and trust, cultural understanding, and context-specific care were key features of effective prenatal care and challenge the usual healthcare model according to the participants.

There is no shortage of qualitative research with Indigenous women indicating relationships with prenatal HCPs are often poor, and women voice regularly experiencing impersonal and dismissive clinic visits as well as judgmental and seemingly untrustworthy HCPs [7–12]. Clearly there is room for improvement to develop positive and trusting relationships with HCPs, which Indigenous women desire [7, 9]. In the group of HCPs we interviewed, they perceived that taking the time to invest in honest/trusting, heartfelt, and friendly interactions was crucial to successful relationships. This mirrors the perceptions of pregnant inner-city women living in a Canadian city (50 % of which were Indigenous), who described valuing HCPs that were trustworthy, non-judgmental, and took the time to make personal connections with patients [13]. Care approaches that are centered on building a respectful relationship with patients have been shown to lead to positive patient experiences, better clinical outcomes, patient compliance with care recommendations, and health-promoting behaviours in a wide range of populations [20–22].

Our findings add to a large and growing body of research calling for greater cultural understanding on the part of prenatal HCPs of Indigenous women [7–12, 23], including the recent World Health Organization recommendations on health promotion for maternal and newborn health [24]. A variety of terms are used in the literature, including cultural safety, awareness, competency, humility, appropriateness, and sensitivity, among others, but the message is the same: HCP cultural understanding of Indigenous women is lacking, and prenatal care and outcomes suffer as a result. HCPs, to be effective, must learn more about the extensive impacts of colonization and the resultant inequities in the social determinants of health, as well as the distinct cultural practices of the Indigenous group they are serving.

Excellent clinical practice guidelines exist aimed at improving the cultural understanding of prenatal HCPs (many of which recommend increasing the numbers of Indigenous HCPs), including recent Canadian consensus guidelines of the Society of Obstetricians and Gynaecologists [25]. However, our findings indicate that reading alone is likely not enough to achieve meaningful cultural understanding, and that it may be strengthened by shared experiences involving HCPs, patients and neighboring Indigenous communities in working together. Such collaborations would allow for mutual learning, real-life experiences, and relationship building, and could be potentially accomplished via attending cultural events and ceremonies, developing workshops led by Elders and/or community members, and involving Elders and/or community members in the clinic environment [26, 27]. More research is needed in Indigenous populations and to assess any impact of HCP cultural competence training on prenatal health status and pregnancy outcomes.

Context-specific care, as described by the participants in our study, requires flexibility and extra time and resources on the part of HCPs to establish relationships and rapport with patients and neighbouring clinics and hospitals, develop a strong cultural understanding, and tailor existing care models to better meet the needs of their patients. Our findings support an ethnography with First Nations women and University academics that found ineffective relationships with HCPs were often the result of structural limitations of the healthcare system [9]. Similarly, a recent qualitative description with prenatal HCPs treating pregnant inner-city women suggests healthcare system barriers (including HCP lack of time, shortage of HCPs) prevent appropriate prenatal care. The authors call for more accessible, convenient, and responsive prenatal care approaches [13]. Our findings also build upon qualitative research calling for equality and shared power in the patient-provider relationship to avoid fear-inducing, paternalistic and ineffective healthcare [7, 12]. Solutions to improving care and strengthening HCP-patient relationships may lie in supporting efforts toward more localized and community-based care, such as Indigenous birthing centres and Indigenous midwifery [10, 25, 28, 29] of which the Inuulitsivik midwifery service and education program in the Inuit region of Quebec, Canada is an excellent example of [30]. Another example of such a program functioned briefly in the same town where some of our research was conducted [31], and ended because of lack of on-going funding. This needs to change, funding for prenatal care is known to derive better pregnancy outcomes [32, 33].

Although Indigenous women often share a similar history of colonization and disparities in the social determinants of health, transferability of the results to other Indigenous populations and communities may be limited as communities are distinct in many ways. Some of the HCPs from surrounding non-Indigenous communities were unable to take part in interviews and we were unable to capture their experience and views. However, the fact that most of our participants were non-Indigenous and had a strong desire to be better HCPs, and hope that others would benefit from their mistakes and experiences, was an encouraging finding. Another limitation of this study is that we did not capture the perspectives of pregnant Indigenous women, only those of HCPs. Our CAC was adamant in allowing the HCPs to have their own voice. We are currently capturing the voice of childbearing women from the community in a parallel qualitative study.

## Conclusions

Prenatal HCPs in Canada are likely to encounter Indigenous patients, and must have better knowledge and cultural expertise to improve this working relationship. Efforts are needed to repair and improve patient-provider relationships, focusing on investing time to develop enjoyable interactions, trust, mutual respect, and shared power with patients. Time invested up front should be foundational and not be seen as “extra”. HCPs should acquire meaningful and positive cultural understanding of the Indigenous peoples they are working with. Reading seminal literature and/or completing online competency courses are important [32] but are not nearly enough. HCPs, their organizations, as well as institutions involved in training of HCPs could work to partner with Indigenous patients and communities to create opportunities for sharing real-life experiences and building positive, ongoing relationships that ultimately improve the integration of HCPs into communities. Any cultural understanding training needs to include components that are specific to the community since all are unique - reinforcing the need for active engagement, collaboration, and mutual learning. Current healthcare systems, clinics, and HCPs need to allow for more innovation, flexibility, and responsiveness in care approaches when working with Indigenous prenatal patients so that care is specific to their context. There is also a need for improved information sharing and coordination of care between hospitals and clinics that provide prenatal and obstetrical care to First Nations women.

Identifying practical ways to put the Calls to Action related to Health from the recent Truth and Reconciliation Commission [34] would go a long way in achieving the implications of our research (identified above) and in advancing the care and health of pregnant First Nations women. Specifically, this would include: acknowledging that current Aboriginal health is a direct result of colonization and of previous government policies (including residential schools); implementing Aboriginal people’s health-care rights as identified in international law, constitutional law, and under the Treaties; respecting and meeting the distinct needs of individual Aboriginal communities; recognizing the value of Aboriginal healing practices and implementing them in practice and in collaboration with Aboriginal healers and Elders; increasing the numbers and retention of Aboriginal HCPs; and providing cultural competency training for all HCPs and healthcare students.

## Abbreviations

CAC, community advisory committee; CBPR, community-based participatory research; HCP, healthcare provider

## Footnotes

1 In Canada, three types of Indigenous peoples (sometimes referred to as Aboriginal peoples) are constitutionally recognized: The First Nations, the Inuit, and the Métis peoples. Each group and each community is distinct in terms of their cultural identities, histories, languages, and so on. The Indigenous population represents approximately 4.3 % of the total Canadian population.

2 Residential schools were a federally funded policy whereby Indigenous children were removed from their homes, families, and culture, and forced into church-run schools aimed at assimilation into the dominant Canadian culture and society. The schools were in existence for over 100 years and have had a profound and continuing negative impact on Indigenous populations [34].
